# Supplementary material for: Discovery of potential imaging and therapeutic targets for severe inflammation in COVID-19 patients
Source: Sci Rep. 2021 Jul 8;11:14151. doi: 10.1038/s41598-021-93743-2 (PMC8266867; doi:10.1038/s41598-021-93743-2)
Supplement: Supplementary file 1 — Supplementary Information 1. [file 41598_2021_93743_MOESM1_ESM.docx]

**Discovery of potential imaging and therapeutic targets for severe inflammation in COVID-19 patients**

Hyunjong Lee^1,2,3^, Jeongbin Park^2^, Hyung-Jun Im^2,4*^, Kwon Joong Na^5*^, Hongyoon Choi^1*^

^1^Department of Nuclear Medicine, Seoul National University College of Medicine, Seoul, Republic of Korea; ^2^Department of Molecular Medicine and Biopharmaceutical Sciences, Graduate School of Convergence Science and Technology, Seoul National University, Seoul, Republic of Korea; ^3^Department of Nuclear Medicine, Samsung Medical Center, Sungkyunkwan University School of Medicine, Seoul, Republic of Korea; ^4^Department of Applied Bioengineering, Graduate School of Convergence Science and Technology, Seoul National University, Seoul, Republic of Korea; ^5^Department of Thoracic and Cardiovascular Surgery, Seoul National University Hospital, Seoul, Republic of Korea

**[Correspondence and Reprint Request]**

Hongyoon Choi, MD., PhD

Department of Nuclear Medicine, Seoul National University Hospital

101 Daehak-ro, Jongno-gu, Seoul, Republic of Korea, 03080

E-mail: chy1000@snu.ac.kr, Tel: +82-2-2072-3347, Fax: +82-2-745-0345

Kwon Joong Na, MD

Department of Thoracic and Cardiovascular Surgery, Seoul National University Hospital

101 Daehak-ro, Jongno-gu, Seoul, Republic of Korea, 03080

E-mail: kjna85@gmail.com, Tel: +82-2-2072-2343, Fax: +82-2-747-5245

Hyung-Jun Im, MD, PhD

Department of Molecular Medicine and Biopharmaceutical Sciences, Graduate School of Convergence Science and Technology, Seoul National University, Seoul, Republic of Korea

Department of Applied Bioengineering, Graduate School of Convergence Science and Technology, Seoul National University, Seoul, Republic of Korea

145 Gwanggyo-ro, Yeongtong-gu, Suwon-si, Gyeonggi-do, Republic of Korea, 16229

E-mail: iiihjjj@gmail.com, Tel: +82-31-888-9187, Fax: +82-31-888-9148

* These authors contributed equally: Hyung-Jun Im, Kwon Joong Na, Hongyoon Choi

**Supplementary Figures**

**
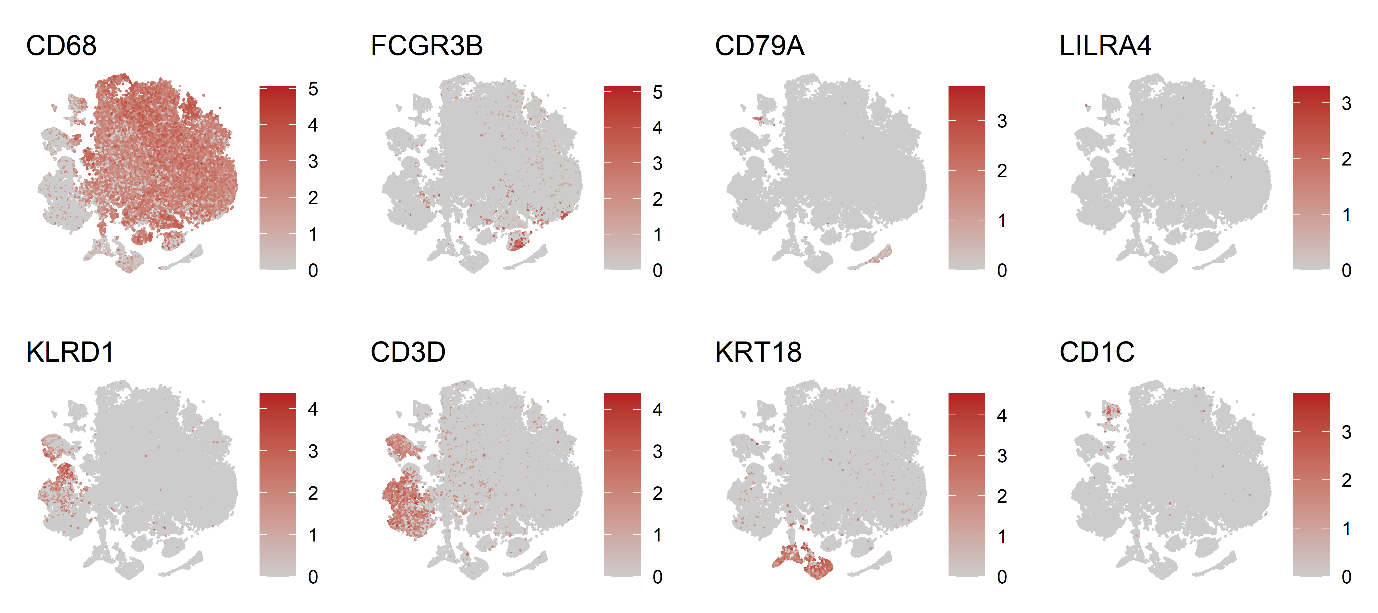
**

**Supplementary Fig. S1 The markers for each immune cell type within BAL fluid.**

t-SNE plots showing the expression of several markers on BAL fluid immune cells; *CD68, FCGR3B, CD79A, LILRA4, KLRD1, CD3D, KRT18,* and *CD1C.*

**
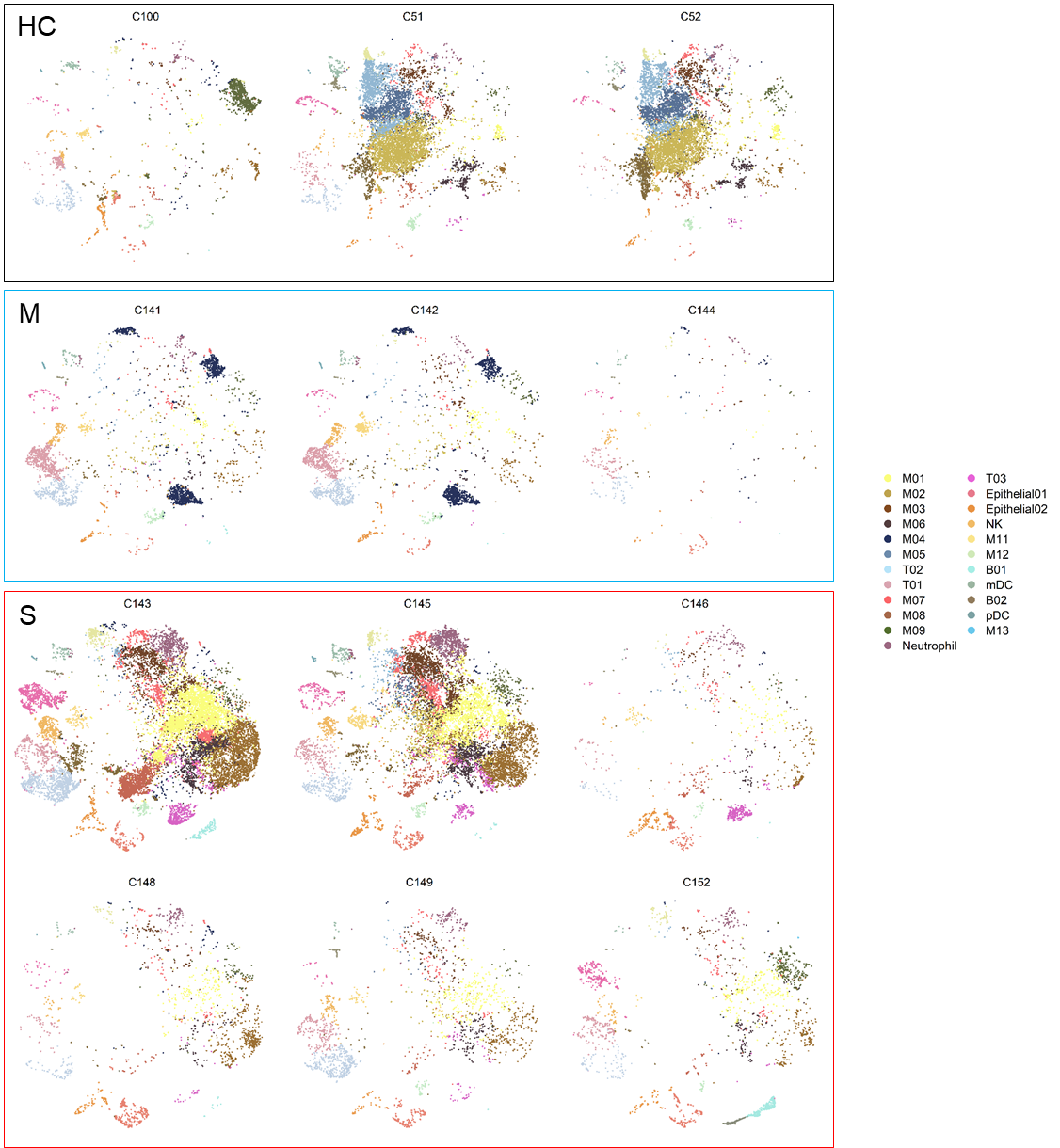
**

**Supplementary Fig. S2 Cell type clustering of each sample in GSE147143.**

t-SNE plots showing distribution of cells in each sample (HC = healthy control; M = moderate COVID-19; S = severe COVID-19).


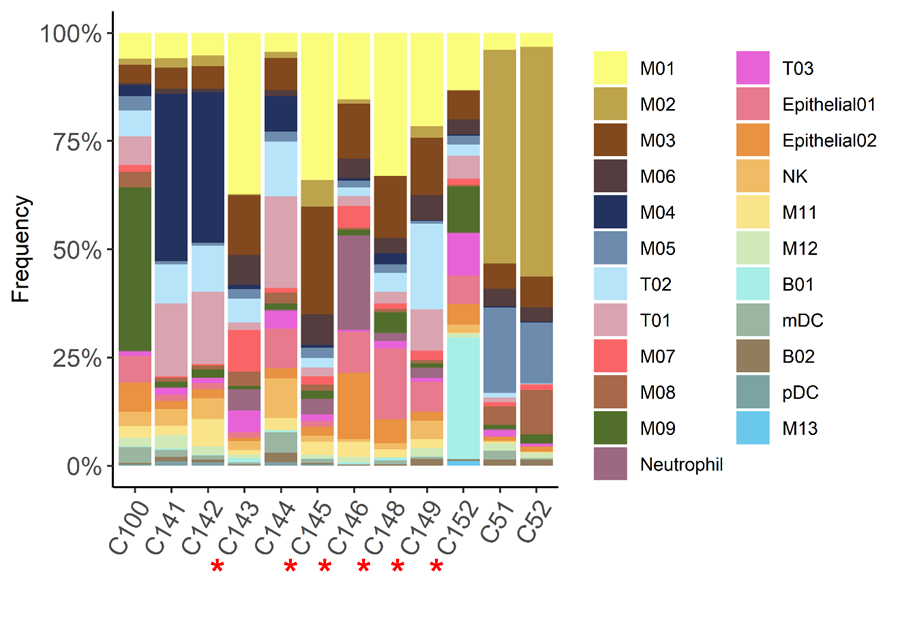


**Supplementary Fig. S3 Cell type population of each sample in GSE147143.**

Barplots showing population of cells in each sample. Samples with red asterisks are severe COVID-19 patients.

**
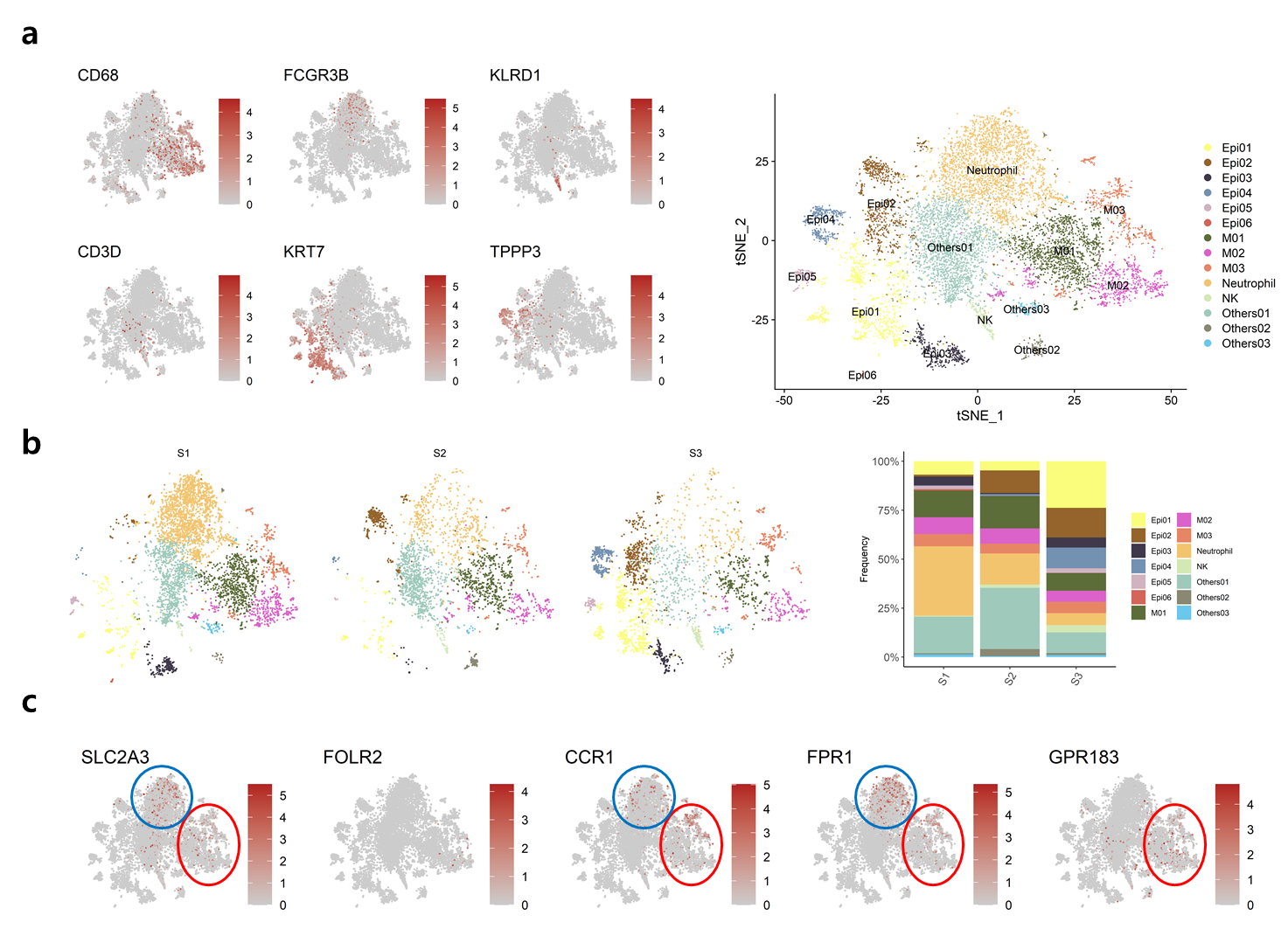
Supplementary Fig. S4 Cell type clustering and expression of candidate molecules in GSE147143.**

**(a)** t-SNE plots showing the markers in GSE147143; *CD68, FCGR3B, KLRD1, CD3D, KRT7,* and *TPPP3 (left).* t-SNE plots showing each cell type *(right)*. **(b)** Distribution and population of cells in each sample was represented in t-SNE plots *(left)* and barplots *(right)*. **(c)** *SLC2A3, CCR1,* and *FPR1* were highly expressed in the macrophage *(red circle)* and neutrophil clusters *(blue circle)*.


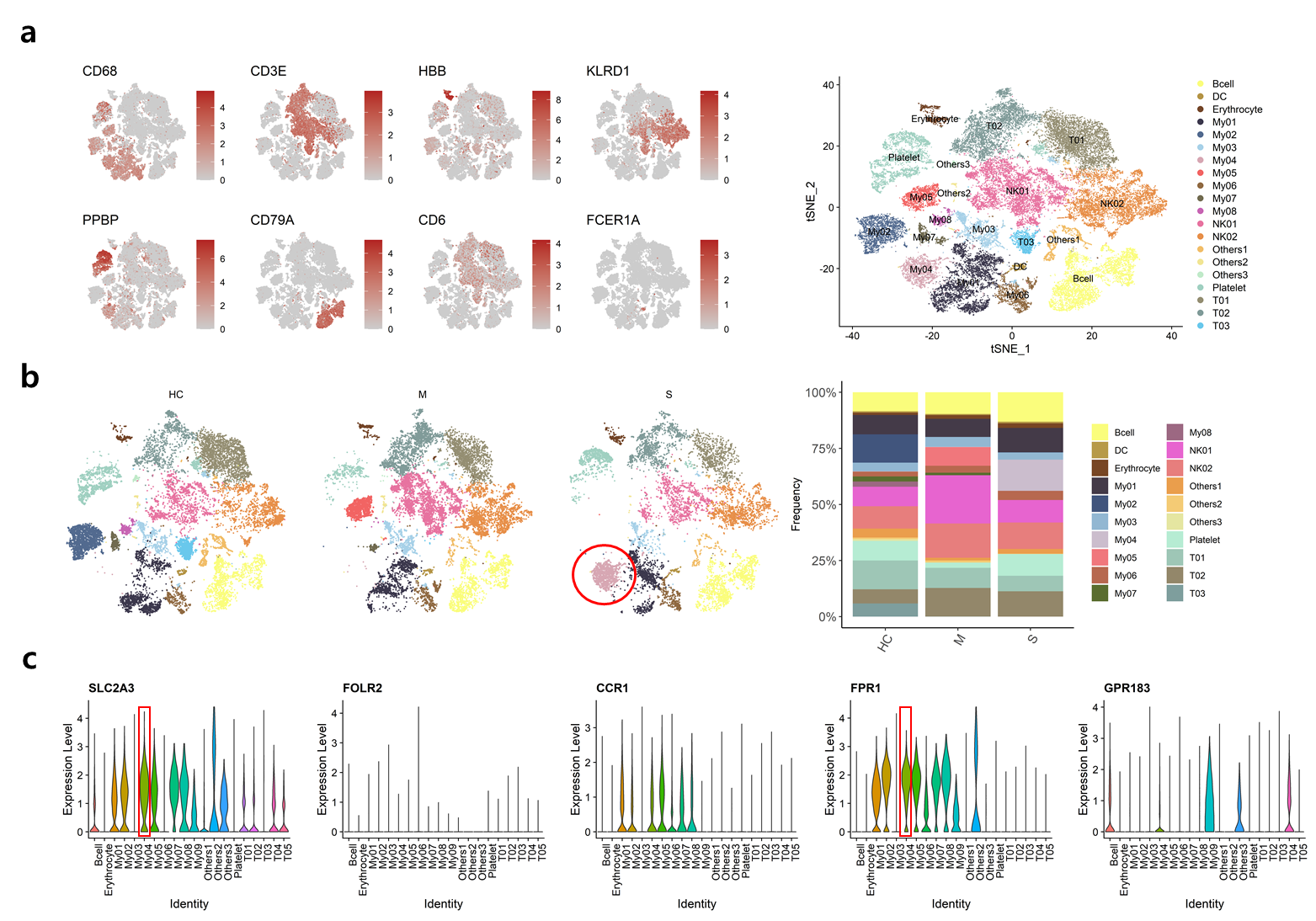


**Supplementary Fig. S5 Cell type clustering and expression of candidate molecules in GSE149689.**

**(a)** t-SNE plots showing the markers in GSE149689; *CD68,* *CD3E, HBB, KLRD1, PPBP,* *CD79A, CD6,* and *FCER1A (left).* t-SNE plots showing each cell type *(right)*. **(b)** There is a specific myeloid cell cluster, My04, in severe COVID-19 patients *(red circle)*. It is also visualized in the barplot *(right)*. **(c)** Expression levels of *SLC2A3*, *FOLR2, CCR1, FPR1* and *GPR183* across cell clusters. *SLC2A3* and *FPR1* were expressed highly in the My04 cluster *(red box)*.


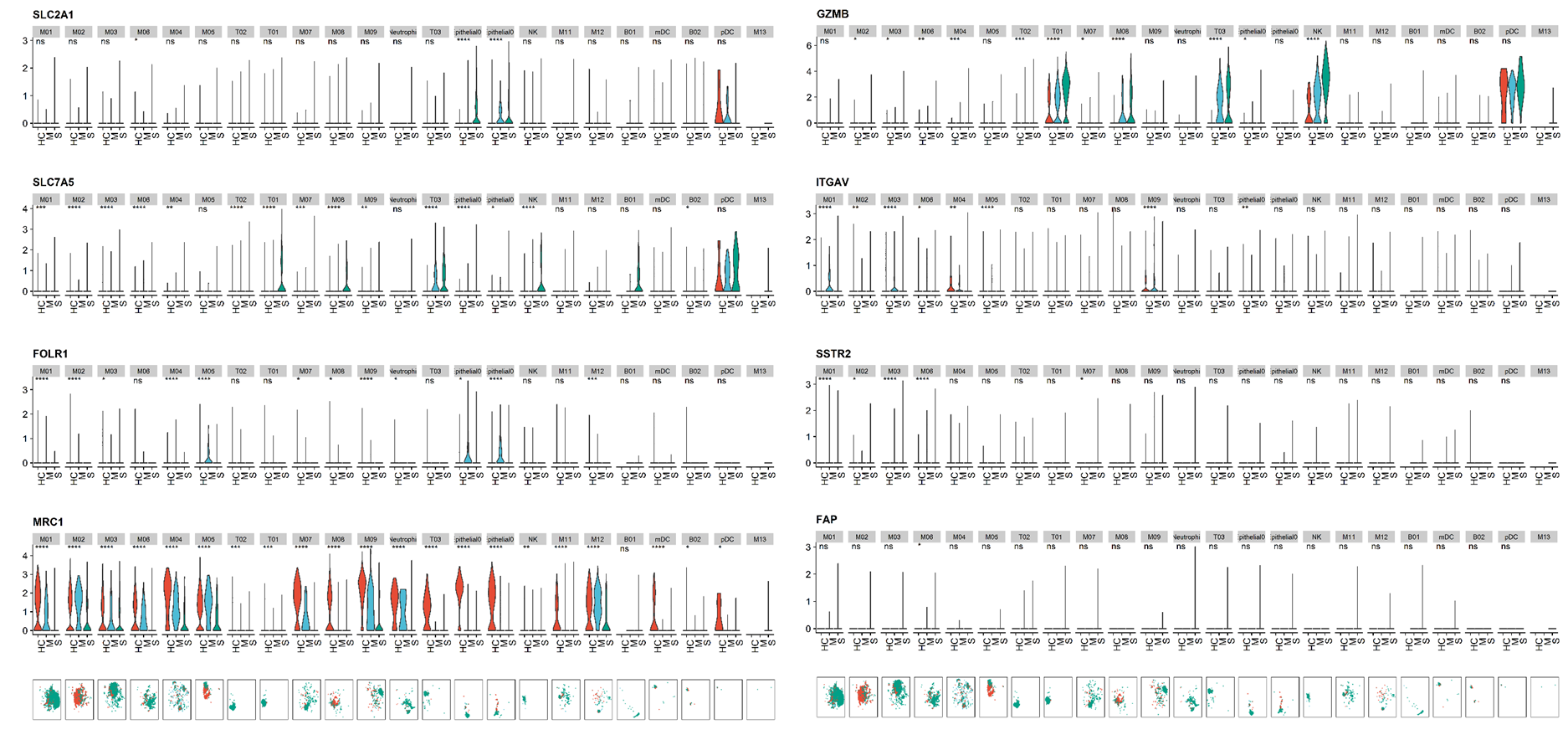


**Supplementary Fig. S6 The expression of alleged imaging markers for inflammation.**

The expression levels of alleged imaging markers were evaluated across immune cell clusters and compared between three groups.; *SLC2A1, SLC7A5, FOLR1, MRC1, GZMB, ITGAV, SSTR2*, and *FAP* (ns: p > 0.05; *: p <= 0.05; **: p <= 0.01; ***: p <= 0.001; ****: p <= 0.0001) (HC = healthy control; M = moderate COVID-19; S = severe COVID-19) t-SNE plots on the bottom panels show distribution of each immune cell cluster. (red dot = HC; blue dot = M; green dot = S).
